# The Impact of Efficacy, Values, and Knowledge on Public Preferences Concerning Food–Water–Energy Policy Tradeoffs

**DOI:** 10.3390/ijerph17228345

**Published:** 2020-11-11

**Authors:** Najam uz Zehra Gardezi, Brent S. Steel, Angela Lavado

**Affiliations:** School of Public Policy, Oregon State University, 300 Bexell Hall, Corvallis, OR 97331, USA; bsteel@oregonstate.edu (B.S.S.); lavadoaa@oregonstate.edu (A.L.)

**Keywords:** environmental values, environmental efficacy, food–energy policy tradeoffs, food security, energy access

## Abstract

Food, water, and energy (FWE) policies often entail contentious tradeoffs. For example, increasing food production may involve irrigation from riparian sources that may adversely impact fisheries habitats, the siting of solar energy on agricultural lands can impact food production, and increasing food production capacity may require pesticides in certain locations, resulting in environmental pollution. Because public preferences are an important component of support for and opposition to FWE policy design and implementation, it is important to understand the correlates of support and opposition to FWE policy tradeoffs. Using survey data from random household surveys conducted in western U.S. states during 2018, this study examined how environmental efficacy, values, and knowledge affected FWE public tradeoff preferences. The findings suggest that these characteristics do affect public FWE tradeoff preferences, with knowledge being a strong driver of support for food production over biofuels, water friendly crops over meat production and conservation over water intensive agriculture. Additionally, environmental efficacy and pro-ecological attitudes drive support for access to safe drinking water and sanitation over food security for a growing population.

## 1. Introduction

Recent studies have highlighted the competing needs for land and water resources in the production of food and bioenergy [1,2]. Rising energy prices and concern over emissions from fossil fuels have led to a shift in the use of some traditional food crops (e.g., soybean, corn) for fuel, resulting in concerns over food security [3]. Moreover, there is concern over the impact of agriculture on the quality and quantity of water available for societal needs [4]. Such tradeoffs have become particularly salient due to global trends in climate change and population growth. Food security, energy access, and environmental degradation are now central concerns in the policy discourse on sustainable resource management.

The past decade has also seen a considerable emphasis on a nexus approach toward achieving sustainable management of food–water–energy (FWE) resources [5,6,7]. In particular, climate change adaptation strategies highlight the need for an integrative framework to balance potential tradeoffs across the three resource sectors [8,9]. Interlinkages across these sectors create competition in resource use such that demand pressures on one sector can change the availability of another resource in another sector. The interconnections between water and energy have received significant attention and there are concerns that the limited availability of fresh water may restrict the type and scale of energy development [10]. At the same time, there is a considerable amount of energy that is needed for obtaining, treating, and distributing potable water to end users. The nexus approach serves to identify further linkages related to food and ecosystems. Current crop production practices are heavily reliant on energy and water resources and the sustainability of ecosystems is a critical dimension that affects all three sectors. Ecosystem degradation can undermine the availability of water and threaten human health while also impacting energy access and food production. However, despite an increasing body of scientific and technical work that documents the interdependencies in the use of nexus resources, there has been scant academic attention to the public receptiveness of policies that entail cross-sectoral tradeoffs [11,12].

In this study, we gauged citizen support for policy tradeoffs around food, water, and energy resources using data from a household survey conducted in four western U.S. states. Three of these—California, Oregon, and Washington—are culturally liberal, “blue” states and proponents of environmental sustainability within state-level policies. The fourth state was Idaho, which served as a comparison case in our analysis of policy preferences. Increasing pressures on existing resources affect the public directly through their implications on food security, clean energy, and water quality, as well as the associated impacts on health and wellbeing. Citizen support is a key component of political acceptability, which is required for the effective design and implementation of a policy, and knowing public preferences helps policymakers understand the potential impact of public policies on different groups of citizens [13]. This study also examined the individual characteristics that drive the support for different policies. The objective was to understand whether characteristics such as efficacy, beliefs, and values can determine how people respond to specific resource tradeoffs. The relationships between food, water, and energy using a public opinion survey have previously been discussed by Portney et al. [11], who examined the public awareness of the linkages across nexus elements. Using the same data, Bullock and Bowman [12] examined the drivers of policy support for managing food, water, and energy resources. However, citizen support has not previously been examined within a framework wherein individual policy options are considered in reference to specific tradeoffs. Moreover, this approach is distinct from previous research that aggregates policy options to create composite measures of policy support [14,15]. In addition, a unique theme that is analyzed in this study is how environmental efficacy, i.e., the feeling that one’s efforts and behaviors can influence policy outcomes, will have an impact on their policy preferences.

## 2. The Food–Water–Energy (FWE) Nexus

There are diverse interactions between the food, water, and energy sectors. Water, for example, is essential in the energy sector for the fossil fuel production chain, biofuel production, and hydropower generation. Similarly, energy is necessary to treat and distribute water. As for the interaction between water and food, water is essential for crop and livestock production and food preparation. Likewise, energy is an important input for agricultural production, such as mechanization for land preparation, irrigation, and fertilizers, and it is necessary to transport and prepare food [7,16]. These interlinkages are distinguished by three specific kinds of interactions: (1) physical, biophysical, and chemical, where the three systems exchange energy and mass; (2) resource inputs and outputs, where each resource is an input and/or an output for the production of other resources; (3) institutions, markets, and infrastructure, where each resource is regulated by different institutions, is shaped by different market dynamics, and requires specific infrastructure. Notably, these interactions can be affected by socioeconomic, technological, and environmental changes [17].

Population growth, natural resources depletion, and climate change are some of the critical factors considered when proposing a novel framework for addressing the threats posed to food, water, and energy security, and to develop innovative and efficient management systems that create greater synergies between these sectors [17,18]. Driven by these goals, the nexus is based on three principles: investing to sustain ecosystems services, creating more with less, and accelerating access by integrating the poorest [7]. To accomplish the FWE nexus goals, it is necessary to develop technical research, economic analysis, and policy formulation all within the lens of the interlinkages of these sectors [7,19]. Specifically, it is necessary for policies to be more integrated and for the decision-making process to be better able not only to boost productivity in the near term but rather more broadly, to encompass efficient resource management over the long term [7]. That is, the nexus approach seeks to elevate concerns of immediate production maximization to a platform driven by more sustainable production [17].

In most studies, the nexus is analyzed as interactions between two sectors (most often food and water) using tradeoff analysis. However, there are a small number of studies where the nexus is analyzed as interactions between the three sectors [20,21]. Johnson et al. [22] estimated that only 4.4% of the modeling studies contemplated the entire FWE nexus. One aspect that adds difficulty to the integration of the three sectors into the nexus analysis is that water prices are embedded in the price of food products and energy services, generating an economic gap between food, energy, and water markets, thus distorting the financial relationships within the nexus. As a result, water mismanagement has prevailed in the agricultural and energy sectors, causing ecosystems to be overexploited. This affects how water is valued in these sectors, which should be considered when devising alternatives for the operation of the nexus [23,24].

Complete operationalization of the FWE nexus requires the consideration of many aspects of each of the sectors that encompass the nexus. In order to balance the tradeoffs and to build effective synergies, it is important to consider multiple interests and diverse stakeholders, which makes the task difficult [17]. Larcom and van Gevelt [25] pointed out that nexus regulation involves multiple levels of governance (local, state, federal, and international entities), as well as non-state actors (community organizations, private companies, and social norms), which create a multitude of complex interactions. There is only a limited amount of research about how to address the integration of the three elements of the nexus and how to implement an integrated approach to optimizing the FWE nexus [20,26]. There are scant examples in the literature that demonstrate policy advances regarding implementing the nexus approach or that examine public support for such approaches [27]. Thus, there is a need to develop more research about how to support decision-making within the nexus approach [21,28]. In addition, the diversity of actors, policies, and institutions and the broad national and regional settings in which FWE nexus policies are implemented make it difficult to generalize the findings and transfer the experience across borders [20]. It was the goal of this study to examine public preferences concerning hypothetical FWE policy tradeoffs and to examine the impact of efficacy, environmental values, and public knowledge on policy tradeoff preferences.

## 3. Values–Beliefs–Norms (VBN) Theory and Correlates of FWE Tradeoffs

Finding what variables demonstrate the potential for stable, consistent actions is critical to formulating public engagement in resource management activities. The VBN theory is a cognitive approach that suggests that individuals will engage in activities that are consistent with their values when they believe their actions can impact the outcome and feel compelled to act based on their values [29]. Additionally, as Stern has argued [30] (p. 409): “Although these behaviors affect the environment only indirectly, by influencing public policies, the effects may be large, because public policies can change the behaviors of many people and organizations at once.”

VBN theory has been used to explain environmental behaviors as an outcropping of values that can be measured using Dunlap et al.’s [31] new ecological paradigm (NEP) scale [32,33,34]. The NEP scale has been successfully utilized as a scale to assess attitudes, beliefs, and values as indicators of a broader endorsement of a worldview that is ecologically centered [35,36,37,38]. These pro-environmental values provide the basis for action when individuals are aware of environmental problems and feel their actions can positively impact the outcome. Therefore, this study used VBN theory as a general frame for examining the impact of efficacy and environmental values on FWE tradeoffs. In addition, various demographic control variables were included in the analyses along with knowledge concerning FWE issues. A brief overview of this literature follows.

### 3.1. Efficacy

According to social cognitive theory [39], perceived self-efficacy is the extent to which people believe themselves capable of undertaking specific behaviors in order to avert a negative outcome or to achieve a certain goal. It has been a dominant concept in health communications research [40,41], and more recently, the literature on climate adaptation has highlighted the notion as being central to understanding human responses to climate change [42,43,44].

Self-efficacy is an important driver of behavior [45] and has been linked to environmental activism and greater public engagement [46]. According to Vancouver et al. [47] (p. 36), self-efficacy is “the most popular form of expectancy belief in the applied psychology literature.” It affects the cognitive appraisal of a stressor by accounting for the perceived severity and the cost of undertaking any action. Individuals will be less prone to act if they believe that required personal action is beyond their ability or is unlikely to resolve a given problem. On the other hand, pro-environmental engagement is associated with whether individuals perceive themselves, and the groups to which they belong, as being capable of coping with large-scale (environmental) problems. In the climate change literature, individuals with higher self-efficacy are reported to be capable of enacting better adaption strategies [44,48]. Moreover, individual perceptions of self-efficacy are positively associated with a sense of collective efficacy. For instance, Jugert et al. [49] report that perceptions of control are elevated when individuals perceive themselves to be part of an efficacious community. Environmental research that calls for the inclusion of civil society and greater citizen engagement [50,51] highlights this implicit connection between self-efficacy and sustainable behaviors.

The beliefs about self-efficacy can also translate into a sense of collective action, which is necessary to address common goods [52] problems such as those associated with the resource nexus. Perceived efficacy has a critical influence on whether the public chooses to engage in political action around an issue [53]. Attitudes toward resource use are often tied to the set of beliefs with which people evaluate their perceived capacity to take actions that prevent resource degradation [54]. To this end, the interplay between knowledge, environmental concern, and perceived efficacy is documented to be an important motivator of public support for climate change policies [43,55,56]. A study by Geiger et al. [57] used an experimental setting to show that knowledge-based interventions influence efficacy beliefs and that these, in turn, catalyze public engagement. A significant concern for an issue such as climate change has been linked to greater perceived efficacy, as well as a higher sense of responsibility to develop solutions [43]. The belief in one’s personal capacity can therefore shape an individual’s interaction with the environment toward the goal of sustainable resource management. McGinty et al. [58] discussed the role of self-efficacy regarding land-use decisions and found a positive association between self-efficacy and the farmers’ intentions to adopt agroforestry. Moreover, a high sense of efficacy is associated with pro-social behavior and the readiness to sacrifice for the benefit of others [59,60].

### 3.2. Values and Beliefs

An extensive body of literature connects social-psychological constructs with general pro-environmental behavior (Gifford and Nilsson [61] provide a review). Studies within environmental sociology have examined the association between individual values and support for pro-environmental policies [6,14,62]. These hold that individuals support policy actions that reflect their value orientations with respect to the environment [63,64].

The impact of values on personal norms and the subsequent acceptability of pro-environment policies is often discussed within the framework of the VBN model [30]. The VBN theory posits that specific responses to environmental issues are influenced by core values and general beliefs. It identifies a process whereby behavior can be predicted by (i) whether an individual believes there to be a threat to personal values and (ii) a perceived ability to reduce the threat (thereby restoring personal values). The latter can be connected to the individual’s assessment of personal efficacy that may interact with values that subsequently drive behavior. Within these models, environmental beliefs are often measured using the NEP scale that was developed by Dunlap et al. [31]. The NEP scale considers an individual’s general environmental beliefs about the biosphere and the effect of humans’ actions on it. It incorporates the notion of “limits to growth” and the balance between human activity and natural resource preservation. Steg et al. [15] found a high score on the NEP scale to be positively influenced by biospheric value orientations and negatively related to egoistic values.

The NEP is considered to have a mediating role in the relationship between values and specific attitudes/behavior [65] and is highly correlated with personal norms [14]. In the policy preference literature, a pro-NEP position has been associated with support for protecting suburban parks [66], reducing greenhouse gas emissions [14], water conservation preferences [67], energy policy preferences [37,68], and preferences for food–water–energy tradeoffs [69].

### 3.3. Knowledge

Knowledge about resource tradeoffs, and specifically the food–water–energy nexus, has been linked to greater policy support for equitable and efficient management of these resources [11,12,69]. The most common measures used in the environmental literature are perceived (i.e., subjective) and assessed knowledge. Stoutenbotough and Vedlitz [70] find that these two measures are inherently distinct and that the latter is associated with a greater concern for climate policies. The distinction between generalized political knowledge and policy-specific knowledge has also been highlighted by Gilens [71], who found the latter to have a strong influence on political decisions.

A number of studies have linked knowledge about policy-specific facts to public preferences [12,67,72]. In a study of Las Vegas residents, Salvaggio et al. [67] found that factual knowledge about drought is significantly associated with water conservation measures. Similarly, support for the adoption of renewables is higher among individuals with knowledge of environmental and energy technology [37]. Moreover, policy preferences about genetically modified foods (GMOs) have also been demonstrated to change with the provision of relevant scientific information [72].

For issues that require some form of risk assessment, the information deficit model provides a strong rationale for communicating scientific information to the public to align public preferences with the prevailing scientific opinion. The model presumes that a lack of knowledge or public understanding of an issue can lead to a divergence between scientific and public opinion [42,73]. Consistent with this, a lack of knowledge is found to be a barrier to climate adaptation in forest management [74]. Bridging the gap between public understanding and expert opinion allows policymakers to gain constituents’ support for evidence-based environmental policies [70,72].

### 3.4. Demographic Control Variables

The demographic control variables that were included in the multivariate analyses included age, gender, education, and income. These control variables are often employed in VBN research involving environmental values, concern, and behavior. Concerning age, many researchers have found that younger people are more likely to express environmental concerns when compared to older generations [31,75,76,77,78]. Another strong predictor of environmental concern is education level. Many studies have found a positive correlation between higher educational attainment and environmental concern [78,79,80]. However, research on how age and education might affect positions on FWE tradeoffs has not been conducted to date. If consistent with this previous research, we should find younger and more highly educated people to favor more environmentally oriented FWE tradeoff options.

Gender and income are additional demographic control variables that were included in the analyses. While some research suggests that these are less consistent predictors of environmental concern [81], a growing number of studies suggest a correlation (although potentially mild or weak). For example, several studies have found that women are more likely to express environmental concerns [34,77,81,82] and that a higher income increases environmental concern [75,83], particularly in countries with overall higher national incomes [83].

## 4. Methods and Data

**Figure 1:**
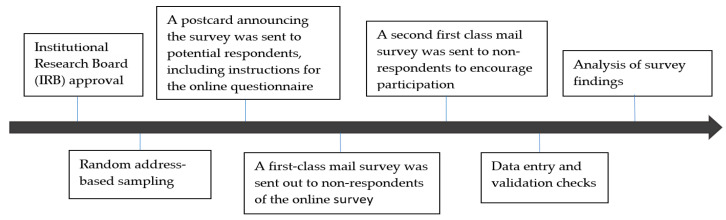
Survey implementation.

This study examined policy preference tradeoffs using survey data from households in four western U.S. states: Oregon, California, Washington, and Idaho. The survey was conducted by the Oregon Policy Analysis Lab (O-PAL) in 2018. Households were selected based on random address-based sampling (ABS) using the U.S. Postal Service’s computerized delivery sequence file (CDS). The CDS includes over 135 million residential addresses, covering nearly all U.S. households. Over 1000 valid residential addresses were generated for each state, resulting in a total of 4695 households being contacted for voluntary participation. Figure 1 provides a snapshot of the stages involved in the implementation of the survey.

The Oregon State University Institutional Research Board approved this research project on 6 December 2017. All student researchers were IRB-certified and worked under the supervision of IRB-certified faculty members. Participation in the household survey was completely voluntary and respondents consented by either following a link to complete the survey online or completing the mail survey and returning it in a business reply envelope. Survey data were stored on password-protected computers.

The survey instrument was designed using the Texas A&M Energy–Food–Water Nexus Public Survey (2015) as a reference but with some modifications, including additional questions on environmental tradeoffs. A modified version of Dillman’s tailored design method [84] was used in a questionnaire format. Potential respondents were sent a mail postcard announcing the survey along with instructions for the online version of the questionnaire (using Qualtrics). This was followed by two waves of first-class mail surveys to non-respondents that encouraged participation. Surveys were distributed via mail between January and March 2018 and each contacted household was issued the following request for participation: “If available, we would prefer the person, 18 years or older, who most recently celebrated a birthday to complete the survey.” Table 1 provides the response rates, which were fairly similar across the states of California, Idaho, Oregon, and Washington (CA = 37.2%, ID = 37.4%, OR = 40.5%, and WA = 38.6%). The percentage of total respondents that used the online Qualtrics survey is reported in the final column (CA = 31.7%, ID = 18.9%, OR = 24.2%, and WA = 19.2%), indicating that the mail survey was the more common modality of response selected by the participants.

Regional surveys have the advantage of being tailored to specific aspects of policy issues that residents are more likely to be familiar with. Public preferences are closely linked to perceptions of events and policies within the surrounding states and responses from regional surveys have important implications for state-level policies. Even as states respond to the global pressures of population growth, urbanization, and climate change, policy action must be consistent with local and regional dynamics surrounding resource degradation and nexus tradeoffs. For instance, while global and national systems are equipped to support biofuel production, regional shortages with regard to land and water can be substantial [85].

**Table 1:** Survey response rates.

State	Surveys	Responses	Response Rate	Online Return Rate
California	1170	435	37.2%	31.7%
Idaho	1175	440	37.4%	18.9%
Oregon	1173	475	40.5%	24.2%
Washington	1177	454	38.6%	19.2%

Note: Response rates were calculated using guidelines from the American Association for Public Opinion Research [86].

## 5. State Case Studies

The climate change impacts on food, water, and energy resources have been experienced across each of the western states included in this analysis. Across these states, frequent flooding affecting water quality in watersheds, substantial loss of snowpack, droughts, and changing temperature threaten the sustainability of current resource processes and have contributed to conflict between resource sectors. For instance, water stress due to droughts and flooding has increasingly focused attention on how watershed ecosystems are being impacted by food production, specifically by agricultural runoff containing fertilizers and pesticides. In Idaho, excess application of fertilizers has been reported to impact groundwater quality in the Eastern Snake Plain Aquifer, which is the source of drinking water for over 300,000 residents of southern Idaho [87]. While fertilizers increase crop yield, the return flow of irrigated water delivers contaminants and sediment to the shallow groundwater system, which impacts the supply of drinking and urban use water.

Contentious debates surrounding hydropower dams and their impact on the fish in Columbia River [88] illustrate yet another example of the food–water–energy tradeoff. Large dams built on the Snake Basin in Washington are a source of electricity and irrigation, but these have also endangered wild salmon populations. Mandates for instream flows to protect fisheries’ habitat impacts water availability for energy production. In California, the hydroelectric Shasta Dam in the Central Valley was removed to support the upstream salmonid habitat, but it led to a substantial decrease in hydropower production and a loss of $57 million annual hydropower revenue [89].

Additionally, there is competition for the use of land and water resources regarding meeting the energy and food needs of the growing populations in these states. There is a displacement of agricultural land due to urbanization, even as farmland is needed to support increased food production. McKinnon [90] highlights how this has created a further source of conflict between Oregon farmers, namely, those that supported limiting the urban use of farmlands and others strongly opposed to governmental regulation over private property. A further tradeoff exists in the use of feedstock, namely, corn, sugar, and soy, for the production of first-generation biofuels. Despite concern over higher food prices, there is support for biofuel production in order to reduce greenhouse emissions. As in the case of bioenergy, most tradeoffs facing the western U.S. states are inextricably tied to the changes in climate, which demand a shift toward more sustainable use of resources.

## 6. Findings: Policy Tradeoffs

The survey conducted for this study included a series of questions that were designed to gauge preferences on policy measures that involve tradeoffs across food, environment, and energy systems. Respondents were presented with a set of policy options, each representing a competing use of available resources. Given two alternatives, respondents could indicate their level of agreement with either policy approach, or else choose to report that they were neutral. In each case, support for a given policy represented preference relative to an alternative use of resources. Previous research by Pierce and Steel [37] used a similar approach for examining tradeoff preferences toward energy policy alternatives. We examined the survey responses using both descriptive and multivariate analyses.

Table 2 shows the coding of the preferences and the distributions of the response across the states for various tradeoff scenarios included in this analysis. The first statement addressed the food vs. energy tradeoff regarding the use of agricultural land. Biofuel and food compete for land and harvest, and while the production of biofuels can lead to a reduction in the imports of fossil fuels and increase energy independence, biofuels also utilize resources that are used to grow food crops, which impacts food supplies and causes food price inflation [3,91]. The distribution of responses showed that a higher percentage of survey respondents supported food production for human consumption versus promoting biofuels. This preference was consistent across all four states, though in California, the number of respondents that agreed (or strongly agreed) with the policy of promoting biofuel production was somewhat higher relative to Oregon, Washington, and Idaho. California is the largest consumer of transportation fuels in the USA and the interest in biofuel production began earlier in California relative to other states. State policies in California, such as the California Low Carbon Fuel Standard (LCFS) [92], promoted the use of biofuels as a means for promoting energy security, sustainability, and a reduction in the carbon intensity of energy sources [93]. Many respondents across the four states, from 26.2% in California to 37% in Washington, also reported being neutral to this food–energy tradeoff. To the extent that neutral responses are representative of public ambivalence toward either option, policymakers have the incentive to maintain the status quo.

In the second statement, respondents were presented with an agricultural tradeoff between livestock feed crops for meat production and growing more water-friendly foods for direct human consumption. Significant water resources are used up for irrigation purposes in the agricultural sector, primarily for food production. Over a third of the respondents in each state indicated that they were neutral about this specific tradeoff, with the highest number of neutral responses coming from Oregon (41%) and Washington (39%). In these two states, preferences were also skewed toward growing water-friendly foods versus maintaining livestock feed crops, particularly in Washington, where 40.3% of the total respondents agreed or strongly agreed with the former policy. In contrast, more respondents in Idaho preferred maintaining meat production levels than those that support diverting resources to crops that can be directly consumed.

The third statement presented a tradeoff scenario between increased food supplies for a growing population using water-intensive plants versus environmentally conservative agriculture that allowed for increased access to safe drinking water and sanitation. A growing population, together with extended periods of drought brought on by climate change, has created substantial pressures on the available fresh water supplies. More than 40% of the respondents in every state were neutral toward this tradeoff, but between the policy options, the preferences were skewed toward the pro-environmental policy option of conserving water rather than pursuing food security through the increased use of water-intensive plants. Relatively fewer respondents—6.3% in California, 7.7% in Idaho, 4.7% in Oregon, and 3.8% in Washington—strongly agreed with the policy approach of increasing the use of water-intensive plants, e.g., rice to feed a growing population.

## 7. Multivariate Analyses

The objective of this analysis was to examine factors that drive public preferences for managing nexus resources. In particular, we assessed whether an individual’s sense of efficacy, knowledge, and ecological values could explain the preferences for elements of the food–energy–environment nexus. To observe the association between the respondents’ characteristics and their preference for specific policy approaches, we used multivariate logistic analysis.

### 7.1. Dependent Variables: Policy Support

Support for specific policies was coded as binary outcomes where agree or strongly agree responses were coded as a value of 1 and 0 if the respondents were neutral or chose the alternative policy option. In each case, we interpreted the outcome not as a simple measure of support for the given policy, but as a preference for the policy relative to a specific nexus tradeoff. Additionally, neutral responses toward given resource tradeoffs were also included as binary outcomes in the analysis. Recent research highlights the substantive nature of neutral responses to attitudinal questions, as well as the importance of examining the dynamics behind such answers [94,95]. Studies have found individual and demographic characteristics to be associated with a tendency for midpoint answers, such as “neither agree nor disagree” [96,97,98].

### 7.2. Independent Variables

Table 3 provides a description of the key independent and control variables included in the analysis. To capture the effect of our main variables of interest, namely, values, knowledge, and efficacy, we used Dunlap’s NEP scale and created a measure of technical knowledge (quiz), as well as an index for environmental efficacy. Additionally, demographic characteristics were found to influence public perceptions [99], as well as policy support [14]. Age, gender, income, and education were therefore included as controls in the regression analysis.

Quiz: Knowledge of highly specific facts and policy-specific information has a significant influence on policy preferences [71]. To construct a measure for environmental knowledge, we used five statements from the survey that each pertained to tradeoffs across elements of the resource nexus. These were intended to measure the technical knowledge of the respondents within a true–false framework. An example statement was: “Using hydraulic fracturing to remove natural gas from the ground uses significant amounts of water.” Survey respondents were asked to indicate whether they believed each statement to be accurate, inaccurate, or whether they “don’t know.” A scale variable called “quiz” was constructed to reflect the number of questions that the participant answered correctly, from 0 = “no questions correctly answered” to 5 = “all questions correctly answered.” The responses to these statements were used to create a composite knowledge variable, which was then included in the analysis to capture the effect of the respondent’s actual rather than self-reported knowledge.

Efficacy: In order to assess environmental efficacy, respondents were asked to report their level of agreement and disagreement (1 = strongly disagree, 2 = disagree, 3 = neutral, 4 = agree, and 5 = strongly agree) with the four following statements: (1) I feel that my own personal behavior can bring about positive environmental change, (2) I would be willing to accept cuts in my standard of living if it helped to protect the environment, (3) I would be willing to support higher taxes if it helped to protect the environment, and (4) I would be willing to sacrifice some personal comforts in order to conserve resources. An additive index was then created with a range of 4 = lowest level of efficacy to 20 = highest level of efficacy. Cronbach’s alpha for the index was 0.804, indicating a high level of internal reliability.

New Ecological Paradigm: A six-item version of the NEP was included in the survey, which asked respondents their level of disagreement and agreement for the following statements: (1) “The balance of nature is very delicate and easily upset by human activities,” (2) “Humans have the right to modify the natural environment to suit their needs,” (3) “We are approaching the limit of people the Earth can support,” (4) “The so-called “ecological crisis” facing humankind is greatly exaggerated,” (5) “Plants and animals have as much right as humans to exist,” and (6) “Humans were meant to rule over the rest of nature.”

The NEP scale by Dunlap et al. [31] has been widely used in recent research as an indicator of environmental values, concern, and attitudes. It provides a measure of beliefs about the relationship between humans and the natural environment while incorporating the notion of “limits to growth” that are inherent to considerations of environmental tradeoffs. The above statements were used to create an index to capture pro-ecological values (items were reverse coded where necessary). Cronbach’s alpha for the six-item NEP was 0.766.

Demographic Control Variables: The demographic control variables included in the multivariate analyses included age in years, a gender dummy variable, formal educational attainment, and household income before taxes. For age, the question used was: “What is your current age in years?” For gender, the question was: “Please indicate your gender,” with response categories of female, male, and prefer not to say. For educational attainment, the question used and response categories provided were: “What is your level of formal education?” 1 = less than high school (grades 1–8), 2 = some high school (no degree), 3 = high school graduate, 4 = some college (no degree), 5 = two year associate college degree (e.g., AA), 6 = college degree (e.g., BA, BS, AB), 7 = some postgraduate schooling (no degrees), and 8 = postgraduate/professional degree (e.g., MA, JD). Finally, for income, the question and response categories were: “Which category best describes your household income (before taxes) in 2017?” 1 = less than $10,000, 2 = $10,000–$14,999, 3 = $15,000–$24,999, 4 = $25,000–$34,999, 5 = $35,000–$49,999, 6 = $50,000–$74,999, 7 = $75,000–$99,999, 8 = $1000,000–$149,999, 9 = $150,000–$199,999, and 10 = $200,000 or more.

## 8. Results

Food Production–Energy Tradeoff: The results of the multivariate analysis for the first policy tradeoff between food and energy are presented in Table 4. As individuals advanced in age, they were significantly less likely to support food production as a tradeoff to seeking energy independence through biofuel production. They were also more likely to be neutral in their response to the energy and food production tradeoff. On the other hand, women were significantly more likely to support food production and significantly less likely to support biofuel production on agricultural land.

The likelihood of promoting the production of either resource as the tradeoff for the other was not influenced by income, which remained insignificant across the two models. However, an increase in the attainment of formal education was positively associated with the likelihood of promoting food production over biofuels. Given the tradeoff, the support for promoting food production rather than biofuel production was also positive and statistically significant among individuals that were more well informed about the FWE nexus based on our quiz measure. Moreover, respondents that scored high on the quiz were less likely to be neutral about this tradeoff.

Given an energy food tradeoff, environmental efficacy and pro-ecological values (according to the NEP scale) were significantly and negatively associated with the support for promoting biofuel production over agricultural land. A sense of efficacy in one’s own behavior together with an ecological paradigm that favored the environment made individuals less likely to support biofuel production, even though it did not increase their likelihood of promoting food production. Of interest in this case was the interaction term, which suggested that between homogeneous groups of individuals varying by a single unit on the NEP scale, an increase in environmental efficacy increased support for energy over food production. The effect of either variable was moderated by the other variable, with the significance of the coefficient indicating that pro-ecological values interacted with beliefs about the efficacy to determine one’s individual preferences across resource tradeoffs.

Food–Water Tradeoff: Maintaining or limiting livestock feed crop as it pertains to the tradeoff between meat production against water-friendly foods is an important aspect of sustainable resource management. The results in Table 5 indicate that age was negatively associated with the preference for limiting feed crops and growing more water-friendly foods. However, older individuals were also more likely to be neutral about such a tradeoff and not statistically more likely to support sustained meat production. Unlike with the energy–food tradeoff, where gender was a significant covariate of policy preference, females did not have a differential preference when considering an agricultural tradeoff. On the other hand, education was significant across all models. Higher levels of formal education made individuals more likely to agree that there should be a reduction in water-intensive plants and less likely to agree that livestock feed crops for meat production should be maintained. Interestingly, the effect of income was opposite to that of education, as higher-income individuals were more likely to support maintaining food (meat) production and less likely to prefer reducing water-intensive feed crops when considering the tradeoff between the two.

Knowledge of the resource nexus, as in the case of education, made individuals more likely to choose a reduction in water-intensive plants when considering a tradeoff with livestock feed crops. However, higher levels of efficacy and a more eco-centric orientation was associated with neutral responses on these agricultural tradeoffs. There appeared to be no significant association between these covariates and the likelihood of agreeing with either policy. As in the case of the energy–food tradeoff, the interaction term suggested that the impact of ecological values on the choice of a neutral response was mitigated by beliefs regarding environmental efficacy. The two variables interacted to decrease the likelihood of an individual choosing to be neutral in their response to this tradeoff.

Food–Environment Tradeoff: Table 6 reports the results of the tradeoff between the use of water-intensive plants to meet rising food demand against increased access to water and sanitation. The negative, albeit weak, coefficient for age suggests that older individuals were less likely to support a reduction in water-intensive plants if the alternative was to meet the food needs of a growing population. Women were also less likely than men to support increased access to safe drinking water and sanitation as a tradeoff to an increased food supply. However, knowledge about the resource nexus and higher formal education were positively associated with the water-conserving policy alternative. Similarly, individuals with a higher sense of environmental efficacy and pro-ecological values were more likely to support limiting water-intensive food sources in favor of improved access to water and sanitation.

Increasing food production through water-intensive plants was negatively associated with income, environmental efficacy, and NEP. Eco-centric values made individuals less likely to support increasing the food supply over water quality and sanitation. As with the agricultural tradeoffs, the interaction term was significant and negatively associated with a neutral response to the food–environmental tradeoff. Higher environmental efficacy increased the likelihood of a neutral response, but this association was offset by the interaction with NEP.

## 9. Discussion

The states of California, Oregon, and Washington have traditionally witnessed significant citizen engagement on issues regarding the environment and climate change. Despite the relatively conservative political culture in Idaho, we found some similarities in preferences across the policy tradeoffs. In each state, there was a greater preference for promoting food production over the use of agricultural inputs for biofuel. Similarly, there was little support for increasing the use of water-intensive plants at the cost of reduced availability of water for drinking and sanitation. Notably, for this particular food–environment tradeoff, a large number of respondents in each state (the highest in Idaho at 47%) reported being neutral. This suggested that while there was concern over water quality, the competing concern over food security for a growing population was also salient among residents of these states. The policy tradeoff where we observed a slight divergence in preferences in the state of Idaho was the food–water tradeoff, whereby the option presented to the respondents was a reduction in feed crop for meat production in favor of more water-friendly foods. In Idaho, the preference for maintaining feed crops for meat production was somewhat higher than its alternative, though the overall pattern in responses was comparable to California. There was no marked pattern of differences in tradeoff preferences that could be attributed to a difference in overall political culture across these states

On the other hand, the results of our multivariate analysis identified various factors that may impact policy support across tradeoffs. The key individual characteristics explored in this study were efficacy, environmental values, and knowledge. We observed the effects of these characteristics on the preferences for policy tradeoffs around the use of food, water, and energy resources. Respondents with higher environmental efficacy were less likely to support the production of biofuels in a tradeoff with food production but were also less likely to support food production over maintaining water quality. Given the hypothetical tradeoff scenario, a sense of environmental efficacy increased the likelihood of a person supporting the policy advocating a reduction in water-intensive plants to ensure greater access to clean drinking water and sanitation. A similar, almost identical pattern of results was found for NEP. Notwithstanding the difference in magnitude, the impact of ecological values on policy support was the same as for efficacy. However, in the food–energy tradeoff, the significant interaction term suggests that with regard to the preference toward biofuels, the effect of each variable was moderated by the other. These results align with the findings of Tabernero and Hernandez [100], which show that intrinsic motivations mediate the effects of efficacy on behavior. Interestingly, a pro-ecological worldview made individuals less likely to support biofuels despite an early discourse in which biofuels have traditionally been presented as a sustainable source of energy [93]. However, it is important to interpret these results strictly within the context of the specific tradeoffs that were presented.

Actual knowledge about elements of the resource nexus was positively associated with pro-environmental policy options, such as support for limiting feed crops to grow more water-friendly foods and reducing water-intensive plants for ensuring water quality. This is consistent with previous studies that suggest that knowledge influences concern about policies affecting water quality among the American public [12]. Knowledge was also linked to a strong preference for food production over the use of agricultural inputs for biofuels. Across all tradeoffs, knowledge was negatively associated with the likelihood of being neutral on the subject. The findings suggest that in line with the knowledge deficit model [73], it is possible to gain support for an evidenced-based policy by effectively communicating and educating the public about relevant and factual information regarding the given policy.

Consistent with the existing literature, we also found a significant association between policy tradeoff preferences and demographic characteristics [14,99]. Higher income was associated with a preference for maintaining feed crops for meat production given the alternative use of agricultural land for water-friendly foods. Similar findings by de Boer and Aiking [101] indicate that affluence is negatively associated with the willingness to reduce meat consumption. Women had a consistent preference for maintaining food production for the given food–energy or food–environment tradeoffs. They were less likely to support a policy of reducing water-intensive plants to conserve water for drinking and sanitation and were more likely to support food production over biofuel production. Gender-specific interactions with elements of the energy–food nexus, and particularly the degree of the direct benefit obtained from a resource, have been found to influence preferences [102]. In particular, this may explain why women were more likely than men to support food production when considering the energy–food tradeoff. Similar to the effect of knowledge, education increased the support for the policy of limiting the growth of feed crops to grow more water-friendly foods for human consumption and for promoting food production on agricultural lands.

## 10. Conclusions

This study examined tradeoff preferences across key elements of the food–water–energy nexus using data from four western U.S. states, namely, California, Oregon, Idaho, and Washington. The challenges of sustainable resource management in the face of rising population demands, increasing urbanization, and climate change makes it pertinent to not only explore policy options but to also examine citizen support for these policies. The findings of this study indicate that individual characteristics shaped tradeoff policy preferences. A sense of environmental efficacy representing the willingness to sacrifice and to change personal behavior for the collective good, value orientation in terms of the relationship between humans and nature, and specific knowledge about the resource nexus were important and consistent measures of public attitudes toward sustainable resource use. In particular, there was evidence that factual knowledge about the nexus elements will impact policy preferences and increase support for a more conservationist approach to resource management.

The analysis of policy preferences within a tradeoff framework had a distinct advantage in that it allowed us to specify some context within which respondents reported their preference. This may improve the consistency of responses, and in some cases, help to identify the source of contention around certain policies. For example, the findings of this study suggest that public support around the development of biofuels may depend on how successful policymakers are at addressing specific concerns around food security. Further studies are needed in order to examine a wider range of tradeoffs and to understand how individual characteristics, such as efficacy and knowledge, would interact with the context and physical surroundings of respondents, e.g., to assess whether support for food production over biofuels is consistent across states, regardless of the severity of energy shortages. In addition, the geographical scope of the study can be expanded to allow for a better understanding of statewide influences.

## Figures and Tables

**Table 2:** Food, energy, and water policy tradeoffs.

***Question:* In recent years there has been considerable attention to tradeoffs between food production, water resources and energy supplies. We are interested in *your views* of what should be done in cases where those impacts are likely to occur. Please circle the number in each case that best represents your opinion. [1 = Strongly Agree; 2 = Agree; 3 = Neutral; 4 = Agree; 5 = Strongly Agree]**					
**State** **Abbreviation**	**Promote biofuel production on agricultural lands for energy independence**			**Promote food production on agricultural lands to feed a growing population**	
	**1 = Strongly Agree**	**2 = Agree**	**3 = Neutral**	**4 = Agree**	**5 = Strongly Agree**
	(%)	(%)	(%)	(%)	(%)
CA	12.1	11.2	26.2	26.6	23.8
ID	6.9	5.7	30.6	28.9	28.0
OR	3.3	3.9	35.5	28.5	28.8
WA	3.4	3.6	37.0	28.2	27.8
Chi-square = 75.33, *p* = 0.000; *n* = 1752					
	**Maintain livestock feed crops for use in meat production**			**Limit the growth of feed crops to grow more water-friendly foods directly for human consumption**	
CA	15.5	17.8	33.0	16.6	17.1
ID	17.8	16.8	35.7	14.5	15.2
OR	12.1	11.6	41.4	14.4	20.5
WA	11.0	9.7	39.0	20.9	19.4
Chi-square = 40.01, *p* = 0.000; *n* = 1763					
	**Increase the use of water-intensive plants (e.g., rice) to feed a growing population**			**Reduce the use of water-intensive plants to increase the access to safe drinking water and sanitation**	
CA	6.3	7.0	41.7	23.5	21.4
ID	7.7	9.3	47.2	18.1	17.7
OR	4.7	5.6	40.0	25.6	24.1
WA	3.8	1.8	42.2	27.5	24.8
Chi-square = 47.38, *p* = 0.000; *n* = 1772					

**Table 3:** Independent and control variables.

Variable Name	Variable Description	Mean (s.d.)
Age	Age in years (range = 18 to 98)	Mean = 51.6, s.d. = 16.83, *n* = 1796
Gender	Gender dummy variable (1 = female, 0 = male)	Mean = 0.504, *n* = 1787
Education	Formal educational attainment (1 = less than high school to 8 = postgraduate degree)	Mean = 4.80, s.d. = 1.46, *n* = 1798
Income	Household income before taxes in 2019 (1 = less than $10,000 to 10 = $200,000 or more)	Mean = 5.88, s.d. = 1.80, *n* = 1772
Quiz	Food, water, and energy quiz (0 = no correct answers to 5 = five correct answers)	Mean = 2.56, s.d. = 1.38, *n* = 1804
Efficacy	Environmental efficacy index (4 = low efficacy to 20 = high efficacy)	Mean = 14.16, s.d. = 3.94, *n* = 1793
NEP	New ecological paradigm scale (6 = low level of support to 30 high level of support)	Mean = 20.73, s.d. = 5.43, *n* = 1782
Interact	Interaction variable for efficacy and NEP (Interact = Efficacy × NEP)	

**Table 4:** Logistic regression estimates for the energy–food production tradeoff.

	Prefer Biofuel Production ^a^	Neutral ^b^	Prefer Food Production ^c^
Variables	Coefficient (SE) Exp(B)	Coefficient (SE) Exp(B)	Coefficient (SE) Exp(B)
Age	−0.009 (0.005) 0.991	0.024 *** (0.003) 1.025	−0.026 *** (0.003) 0.975
Gender	−0.471 ** (0.177) 0.624	−0.130 (0.113) 0.878	0.337 ** (0.106) 1.401
Education	−0.027 (0.063) 0.974	0.068 * (0.032) 0.861	0.167 *** (0.038) 1.182
Income	−0.035 (0.050) 0.966	0.068 * (0.032) 1.070	−0.053 (0.031) 0.948
Quiz	0.080 (0.063) 1.083	−0.340 *** (0.042) 0.712	0.247 *** (0.039) 1.280
Efficacy	−0.365 *** (0.067) 0.694	0.275 *** 0(.059) 1.317	0.091 (0.050) 1.095
NEP	−0.343 *** (0.048) 0.710	0.234 *** (0.037) 1.264	0.033 (0.032) 1.034
Interact	0.017 *** (0.004) 1.017	−0.015 *** (0.003) 0.986	−0.001 (0.002) 0.999
*n*	1732	1732	1732
Chi-square	236.938 ***	198.225 ***	222.311 ***
Percent predicted	87.9	68.7	54.0
Nagelkerke *R*^2^	0.245	0.152	0.161

Note. Standard errors (SEs) are reported in parentheses; Exp(B) is the odds ratio reported below the standard errors. ^a^ 1 = agree with promoting biofuel production, 0 = else; ^b^ 1 = neutral, 0 = else; ^c^ 1 = agree with promoting food production, 0 = else; * *p* ≤ 0.05, ** *p* ≤ 0.01, *** *p ≤* 0.001.

**Table 5:** Logistic regression estimates for the agricultural tradeoff.

	Prefers Maintaining Livestock Feed Crops ^a^	Neutral ^b^	Prefers Limiting Feed Crops to Grow More Water-Friendly Foods ^c^
Variables	Coefficient (SE) Exp(B)	Coefficient (SE) Exp(B)	Coefficient (S) Exp(B)
Age	−0.001 (0.004) 0.999	0.009 ** (0.003) 1.009	−0.016 *** (0.003) 0.984
Gender	−0.018 (0.119) 0.982	0.011 (0.107) 1.011	0.009 (0.116) 1.009
Education	−0.136 *** (0.043) 0.872	−0.147 *** (0.039) 0.863	0.304 *** (0.042) 1.356
Income	0.199 *** (0.036) 1.221	−0.075 * (0.031) 0.928	−0.124 *** (0.034) 0.883
Quiz	−0.007 (0.043) 0.993	−0.224 *** (0.039) 1.165	0.275 *** (0.043) 1.316
Efficacy	−0.035 (0.052) 0.996	0.153 ** (0.051) 1.165	0.026 (0.065) 1.027
NEP	−0.060 (0.034) 0.942	0.153 *** (0.032) 1.166	−0.033 (0.043) 0.961
Interact	−0.002 (0.003) 0.998	−0.010 *** (0.002) 0.990	0.006 * (0.003) 1.007
*n*	1732	1732	1732
Chi-square	189.326 ***	118.421 ***	353.053 ***
Percent predicted	74.1	67.0	75.1
Nagelkerke *R*^2^	0.150	0.090	0.255

Note. Standard errors (SEs) are reported in parentheses; Exp(B) is the odds ratio reported below the standard errors. ^a^ 1 = maintain livestock feed crop for meat production, 0 = else; ^b^ 1 = neutral, 0 = else; ^c^ 1 = limit growth of feed crops to grow more water-friendly foods, 0 = else; * *p* ≤ 0.05, ** *p* ≤ 0.01, *** *p ≤* 0.001.

**Table 6:** Logistic regression estimates for the food–environment tradeoff.

	Prefers Maintaining Water-Intensive Plants ^a^	Neutral ^b^	Prefers to Reduce Water-Intensive Plants ^c^
Variables	Coefficient (SE) Exp(B)	Coefficient (SE) Exp(B)	Coefficient (SE) Exp(B)
Age	0.008 (0.005) 1.008	0.000 (0.003) 1.000	−0.007 * (0.003) 0.993
Gender	0.158 (0.180) 1.171	0.201 * (0.104) 1.223	−0.289 *** (0.109) 0.749
Education	−0.094 (0.065)	−0.020 (0.037) 1.085	0.081 * (0.039) 1.085
Income	−0.120 * (0.051) 0.887	−0.084 ** (0.030) 0.920	0.128 (0.032) 1.136
Quiz	−0.029 (0.063) 0.971	−0.093 ** (0.037) 0.911	0.097 ** (0.039) 1.253
Efficacy	−0.163 * (0.070) 0.850	0.154 *** (0.048) 1.166	0.226 *** (0.068) 1.253
NEP	−0.128 ** (0.044) 0.880	0.072 * (0.030) 1.075	0.184 *** (0.044) 1.202
Interact	0.002 (0.004) 1.002	−0.009 *** (0.002) 0.991	−0.006 * (0.003) 0.994
*n*	1732	1732	1732
Chi-square	218.584 ***	77.597 ***	322.409 ***
Percent predicted	89.1	57.2	69.5
Nagelkerke *R*^2^	0.235	0.059	0.227

Note. Standard errors (SEs) are reported in parentheses; Exp(B) is the odds ratio reported below the standard errors. ^a^ 1 = agree with an increase in water-intensive plants to feed a growing population, 0 = else; ^b^ 1 = neutral, 0 = else; ^c^ 1 = agree with reducing water-intensive plants for water quality, 0 = else; * *p* ≤ 0.05, ** *p* ≤ 0.01, *** *p* ≤ 0.001.
